# Adherence type impacts completion rates of frequent mobile cognitive assessments among older adults with and without cognitive impairment

**DOI:** 10.21203/rs.3.rs-3350075/v1

**Published:** 2023-09-29

**Authors:** Kieffer Christianson, Meha Prabhu, Zachary T Popp, Md Salman Rahman, James Drane, Marissa Lee, Corinna Lathan, Honghuang Lin, Rhoda Au, Preeti Sunderaraman, Phillip H Hwang

**Affiliations:** University of Nevada; Boston University School of Medicine; Boston University School of Medicine; Boston University School of Medicine; AnthroTronix (United States); AnthroTronix (United States); AnthroTronix (United States); University of Massachusetts Medical School; Boston University School of Medicine; Boston University School of Medicine; Boston University School of Public Health

## Abstract

**Background:**

Prior to a diagnosis of Alzheimer’s disease, many individuals experience cognitive and behavioral fluctuations that are not detected during a single session of traditional neuropsychological assessment. Mobile applications now enable high-frequency cognitive data to be collected remotely, introducing new opportunities and challenges. Emerging evidence suggests cognitively impaired older adults are capable of completing mobile assessments frequently, but no study has observed whether completion rates vary by assessment frequency or adherence type.

**Methods:**

Thirty-three older adults were recruited from the Boston University Alzheimer’s Disease Research Center (mean age = 73.5 years; 27.3% cognitively impaired; 57.6% female; 81.8% White, 18.2% Black). Participants remotely downloaded and completed the DANA Brain Vital application on their own mobile devices throughout the study. The study schedule included seventeen assessments to be completed over the course of a year. Specific periods during which assessments were expected to be completed were defined as subsegments, while segments consisted of multiple subsegments. The first segment included three subsegments to be completed within one week, the second segment included weekly subsegments and spanned three weeks, and the third and fourth segments included monthly subsegments spanning five and six months, respectively. Three distinct adherence types – *subsegment adherence*, *segment adherence*, and *cumulative adherence* – were examined to determine how completion rates varied depending on assessment frequency and adherence type.

**Results:**

Adherence type significantly impacted whether the completion rates declined. When utilizing *subsegment adherence*, the completion rate significantly declined (p = 0.05) during the fourth segment. However, when considering completion rates from the perspective of *segment adherence*, a decline in completion rate was not observed. Overall adherence rates increased as adherence parameters were broadened from *subsegment adherence* (60.6%) to *segment adherence* (78.8%), to *cumulative adherence* (90.9%).

**Conclusions:**

Older adults, including those with cognitive impairment, are able to complete remote cognitive assessments at a high-frequency, but may not necessarily adhere to prescribed schedules.

## 1 ∣ Introduction

Alzheimer’s disease (AD) is characterized by an insidious onset with progressive neurodegeneration occurring decades before overt clinical symptoms appear^1-3^. The traditional approach for characterizing these symptoms consists of a single session of intensive neuropsychological (NP) testing to determine whether two or more cognitive domains, typically episodic memory and executive function, have declined^4,5^. This approach provides reliable estimates of disease at later stages, but does not capture the subtle symptoms that arise sporadically during the early stages of cognitive impairment (CI), in part because standardized NP assessments cannot be repeated frequently^6^. The subjective experience or observation of these early stage symptoms can be the very basis for pursuing formal cognitive evaluation, that in turn is not structured to detect them^7^. During these milder stages of CI, it is often family members or other informants who recognize occasional displays of behavior as irregular relative to an individual’s normative functioning^8-10^. Daily caretaker reports often loosely operationalize behavioral deviations as resulting in an increased frequency of “bad days”^11,12^, although informant reports often do not correspond with an individual’s self-report^13^. A bad day interpreted in the context of an otherwise good week can be considered a fluctuation, whereas a steady increase in bad days over the course of a year represents a gradual decline. A single session of NP testing conducted on a bad day may result in a diagnosis of mild cognitive impairment (MCI), thus resulting in diagnostic bias. Depending on the study design this may also explain why the reversion from MCI to normal is fairly common^14^.

To distinguish between fluctuations and true decline, frequent assessment of cognition and behavior is required to avoid relying on data generated from a single session. Remote digital data collection now addresses the juxtaposition between subjective longitudinal observation and objective single session assessment by enabling objective cognitive data to be collected longitudinally and remotely. Mobile device usage among older adults has increased over the last several years, as 84% of U.S. adults aged 50+ own a smartphone, and 76% report relying on technology to stay connected with friends and family according to a 2021 survey^15^. Completing assessments at home, rather than in a formal clinical setting may also reduce the risk of the “white-coat effect”, which explains reduced memory performance in a clinical environment^16^. Additionally, allowing individuals to complete tasks on their own device as opposed to a study-issued device may contribute to improving the ecological validity of remote assessments^17^.

Adherence to prescribed study schedules consisting of repeated unsupervised assessments represents a novel element of remote study designs, particularly among older adults with CI who may have difficulties remembering to complete assessments according to schedule. Prior studies support the feasibility of cognitively unimpaired adults completing repeated unsupervised assessments via mobile-platform^6,18-30^. However, evidence as to whether older individuals diagnosed with CI are capable or interested in completing frequent unsupervised mobile assessments remotely is limited^31,32^. Furthermore, no published studies that we are aware of has considered how assessment frequency or adherence type impacts completion rates. These factors represent novel aspects to the evolving practice of self-administered assessments from which agreed upon clinical interpretation standards have yet to be established.

The limited research examining adherence and assessment frequency as it relates to cognitive performance may persist for two reasons. The first is that the NP approach traditionally considered cognitive performance to be a relatively stable entity. However, the field now acknowledges that considerable within-person variability may occur when NP tests are repeated over a short period of time^33^. If cognitive performance is conceptualized as a dynamic process, rather than a stable trait, the timescale of assessment becomes important^34^. Identifying the appropriate assessment frequency to correspond with the temporal dynamics of behavior has been discussed among other populations^35^, but to our knowledge has not been studied in the context of CI. The second possible reason that few studies have examined how assessment frequency impacts cognitive performance could be due to concerns of the ability of older adults with CI to complete unsupervised mobile assessments. Although the feasibility of older adults with CI to complete cognitive assessments at a high-frequency for a period of one-to-two weeks has been demonstrated^31,32^, no study has assessed whether completion rates remain high when assessments are prescribed continuously over a longer period of time. Therefore, this study aimed to determine the feasibility of self-administered mobile cognitive assessments prescribed at varying frequencies among older adults with and without CI using the DANA Brain Vital application over a period of one year.

## 2 ∣ Materials and methods

### 2.1 ∣ Study Sample

The study sample consisted of participants who were already enrolled in a parent study at the Boston University Alzheimer’s Disease Research Center (ADRC). Recruitment occurred via phone or email, and the brief screening agreement was completed electronically while on the phone with a study staff member. Inclusion criteria included not meeting criteria for dementia, owning a mobile device (e.g., smartphone, tablet) with access to the internet, speaking English as a primary language, and being over the age of 40 years at the time of enrollment. Cognitive impairment status was assigned to individuals with MCI according to the National Alzheimer’s Coordinating Center (NACC) Uniform Data Set (UDS) diagnostic criteria version 3^36,37^, or who scored one-and-a-half standard deviations below the normative mean on NP testing in the absence of functional impairment. Among potential participants who were contacted but did not enroll, eleven were uninterested and two did not meet inclusion criteria.

### 2.2 ∣ DANA Brain Vital

#### 2.2.1 ∣ Application Description

The DANA Brain Vital tool was utilized as the mobile application in this study. DANA is an FDA-cleared computerized neurocognitive test accessible as a mobile application for iOS and Android devices. Previous work has demonstrated DANA’s internal consistency and reliability^38^, as well as its sensitivity to cognitive dysfunction specific to AD^39^. The duration of a single assessment session is approximately five minutes and consists of three subtests intended to measure reaction time, executive functioning, and inhibitory control. The subtests are presented in a fixed order and performance feedback is not provided to the participant. The first subtest, Simple Reaction Time (SRT), measures the latency between when a large target appears on the screen and when the participant taps the target across 40 trials. The second subtest, Procedural Reaction Time (PRT), involves one of four numbers (2, 3, 4, or 5) being displayed for two seconds and requires the participant to tap the corresponding left button (2 or 3) or right button (4 or 5) across 32 trials. The third subtest, Go/NoGo (GNG), requires the participant to either tap or omit a response based on the color of stimuli that appear in varying location across the screen across 30 trials^40^.

#### 2.2.2 ∣ Data Quality

Data collected from the DANA application is automatically transferred to a secure web portal accessible to the study staff in raw and derived formats. Raw data consists of specific values for each finger tap completed across every trial, whereas summary scores for each subtest within an assessment constitute the derived outcomes that are intended to be readily interpretable. Any subtest with a percentage accuracy of less than 70% is flagged as an indication that the instructions may not have been comprehended or a distraction may have occurred. An additional feature to mitigate extraneous factors influencing test performance is that the application automatically closes if a phone call is received while the application is open. If the application is reopened within three minutes after closing, the assessment may be continued beginning at the subtest that was in progress prior to the interruption. After three minutes of being closed, the data is discarded and no partial results are recorded.

### 2.3 ∣ Remote Study Design

Participants were given the option to complete the remote study visit via Zoom videoconferencing or phone. After obtaining informed consent electronically, participants were directed to download the mobile application to their mobile device and login using a unique study ID. A practice session using the application was completed to ensure that participants were comfortable completing the assessment tasks. Study staff monitored incoming practice data to confirm assessment task instructions were followed by participants.

**Figure 1:**
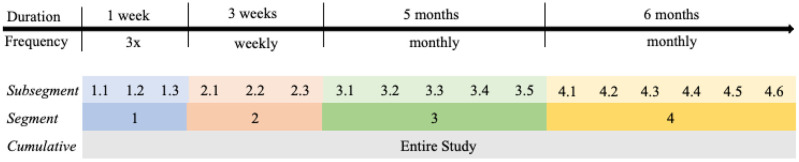
12-Month Study Schedule

A self-administered assessment schedule consisting of four segments spanning one year in duration was provided to each participant (Figure 1). Each segment consisted of multiple subsegments, which were defined as specific periods during which individual assessments were expected to be completed. The first segment included three subsegments to be completed within one week; the second segment included weekly subsegments and spanned three weeks; and the third and fourth segments included monthly subsegments spanning five and six months, respectively. Segment durations were varied to inform whether adherence varied as a function of prescribed segment length. A $25 gift card was mailed to participants for each segment completed on-time and in its entirety. Participants were only asked to complete the minimum assessment schedule, but were not prevented from completing additional assessments. Assessment reminders were provided via text, email, or phone call reminders, depending on participant preference.

### 2.4 ∣ Adherence

Adherence rates were examined using three distinct definitions of adherence that were created for this study to compare how adherence might vary depending on how it was defined.*Subsegment adherence* refers to whether the correct number of assessments were completed precisely within the subsegment dates provided to the participant. Based on this definition, an assessment completed one day after a prescribed subsegment period would render the entire segment as non-adherent. *Segment adherence* only considers the expected number of assessments across the entire segment, disregarding the specific subsegments. For example, a participant who did not compete an assessment during subsegment 2.1, but completed two assessments during subsegment 2.2 the following week, and one assessment during subsegment 2.3 would be considered adherent for segment 2 using the *segment adherence* definition (three assessments within three weeks),but non-adherent using the more stringent *subsegment adherence* (one assessment each week for three weeks)definition. Both *subsegment* and *segment* adherence were evaluated individually across each segment and collectively across the overall study. To meet the overall criteria within either the *subsegment* and *segment* adherence type, segment adherence across each individual segment was required. Finally, *cumulative adherence* considers the total number of assessments completed during the entire study period, irrespective of specific segment or assessment period deadlines. These different definitions of adherence were examined to determine how the temporal resolution of assessment frequency impacts feasibility. Within the scope of this study, the progressively relaxed definitions for adherence provide different insights as to whether participants completed the total number of scheduled assessments, and separately whether they adhered to the specific schedule.

### 2.5 ∣ Analysis

Descriptive statistics for the sample demographics were provided as means and standard deviations for continuous variables or counts and percentages for categorical variables. Adherence rates were computed by dividing the number of participants characterized as adherent within each definition by the total number of participants. Cochran’s Q test was used to evaluate whether there were significant differences in adherence across assessments. Pairwise McNemar's chi-squared testing with continuity correction was used for post-hoc testing to compare adherence rates between all segments within the *subsegment* and *segment adherence* definitions. Statistical significance was based on two-sided p<0.05. All statistical analyses were conducted in R version 4.2.2 (R Foundation for Statistical Computing; Vienna, Austria).

## 3 ∣ Results

The sample consisted of older adults who were predominantly female, white, well-educated, and cognitively unimpaired (Table 1). Most participants in the study used iPhone mobile devices to complete the cognitive assessments in DANA.

Utilizing the strict *subsegment adherence* definition, the completion rate declined during the study (93.9%, 90.9%, 81.8%, and 72.7% for segments 1-4, respectively). When applying the broader *segment adherence* definition, the adherence rates remained relatively high across segments (93.9%, 97.0%, 97.0%, and 87.9% for segments 1-4, respectively). Significant differences were observed across segments when comparing across the *subsegment adherence* completion rates (p=0.04), but not for the *segment adherence* completion rates (p=0.35). Within the *subsegment adherence* definition, the segment 4 adherence rate of 72.7% significantly differed from segment 1 completion rate of 93.9% (p=0.05) (Table 2). Overall completion rates (Table 2) increased as adherence parameters were broadened from *subsegment adherence* (60.6%), to *segment adherence* (78.8%), to *cumulative adherence* (90.9%).

## 4 ∣ Discussion

### 4.1 ∣ Adherence Type and Completion Rates

Our study supports the growing literature that older adults, including those with CI, are capable of completing high-frequency, mobile-based cognitive assessments. We observed that the stringency by which adherence is defined may also influence completion rates in our sample. This is relevant to the emerging practice of self-administered assessments in that individuals have the latitude to choose when to complete assessments at home or in any quiet environment with internet access rather than during a scheduled time in a clinical environment. Within this present study, the progressive discrepancy between the overall rates for *subsegment adherence*, *segment adherence*, and the *cumulative adherence* indicates that many individuals completed the total number of prescribed assessments, but that they did not complete them according to specific deadlines. In other words, as adherence criteria is relaxed from using specific subsegment dates to considering broader segment dates or the cumulative study duration, participants continued to complete assessments at a consistent rate over a span of one year. This discrepancy between adherence type provides an initial perspective towards how self-administered completion rates may vary based on frequency such that individuals were less adherent to the specific study schedule as segment durations lengthened and assessment frequency decreased. Also, the *overall subsegment adherence* rate as compared to the *specific subsegment adherence* rates indicates that different participants were non-adherent across different segments, suggesting the phenomena was not isolated to select individuals.

This study supports the feasibility of the high-frequency assessments among older adults, including those with CI, which was previously reported in studies with shorter assessment periods. Among the emerging literature, Nicosia et al., (2023) asked participants to complete up to four brief mobile cognitive assessments per day across three seven-day periods spaced six months apart, and found no difference based on CI status and observed an overall adherence rate of 80.42%. Cerino et al., (2021) included up to six daily assessments for 16 days, and found only a slight difference in adherence based on CI status wherein the mean completion rate for cognitive unimpaired adults was 85.20%, while the rate for adults with MCI was 78.10%. Ours is the first study to span a complete year with continuous assessments and to compare completion rates according to different assessment frequencies and adherence types. Defining specific adherence types not only provides insight in terms of how assessment frequency can influence completion rates, but also provides a framework to consider how completion patterns of self-administered assessments could provide diagnostic utility.

### 4.2 ∣ Enabling Process Based Detection of Cognitive Impairment

The traditional approach to detecting CI relies on identifying intra-individual variability or *dispersion* (IIV-D) across cognitive domains^42-45^. This approach compresses item-level responses into subtest scores that are then converted into standard scores to decipher whether significant discrepancy (i.e., dispersion) is observed across cognitive domains. No information regarding the strategies or processes utilized to answer specific question is accessible using this method, and no patterns in responses can be identified. The *Boston Process Approach (BPA)* was pioneered to address this shortcoming by emphasizing process-based scoring (e.g., characterizing cognitive error types to differentiate disease pathology that would otherwise be indistinguishable using traditional NP summary scores)^46^. Modern adoptions of a process-based approach have demonstrated the utility in coupling granular digital data with advanced analytics to uncover novel indices of cognitive functioning^24,46-51^. For example, learning effects observed during repeated cognitive assessments are typically interpreted as confounds to valid test interpretation. Yet recent process-based evidence suggests that an absence of a learning effect after repeated assessments is associated with amyloid beta positivity in cognitively unimpaired adults at risk for cognitive decline^24,51^. This granularity afforded by digital cognitive data enables the ability to consider whether fluctuations across or within assessments, or intra-individual variability in *inconsistency* (IIV-I)^52^, that would traditionally be considered “noise” might be a meaningful signal^34,53^. Despite decades of research demonstrating the diagnostic association between inconsistency in cognitive performance (IIV-I) and CI in controlled research settings using laboratory computers^31,54-60^, the transition to utilizing unsupervised mobile devices for remote data collection remains in its infancy^61^. The DANA Brain Vital application utilized in this study exemplifies the possibilities afforded by digital data collection. For example, whereas a traditional reaction time test (and many computerized reaction times test today) provide only a mean single reaction time value, the DANA SRT subtest provides 30 intra-trial values from which a single mean value is derived. This granularity of data not only enables the total amount of IIV-I be measured, but it enables the patterning of variability to also be accounted for^62^.

Even among the limited use of process-based approaches currently utilized in research literature, all associate some metric cognitive performance with meaningful clinical or neurological outcomes. Within the context of high-frequency cognitive assessments to detect the presence of cognitive decline, it may be assumed the only utility in monitoring adherence would only be to ensure enough assessments are completed to detect meaningful changes in objective cognitive performance. However, considering adherence patterns as a separate variable relevant to the onset of CI could represent an expansion of the BPA that considers aspects of behavior beyond traditional cognitive performance.

### 4.3 ∣ Accelerated Progress Forward

Prior to COVID-19 necessitating the transition to remote cognitive assessment, some reluctance to embrace technological assessment approaches persisted among neuropsychologists^49,63^. The term Hybrid Neuropsychology has since been introduced as a model to modernize the field by integrating technology, data science, and engaging with innovators in other fields^64^. This transition forward is largely contingent on the ability to collect both cognitive and lifestyle data remotely and repeatedly, which may enable populations with limited access to resources based on sociodemographic and/or geographic factors to participate in research and reduce reliance on population-based norms which can be biased based on educational and cultural context^65,66^. In doing so, the traditional approach of considering how between-person variables (i.e., age, disease status) impact within-person processes (i.e., cognitive functioning)^67^ can evolve into acknowledging the myriad of within-person factors (i.e., daily activities, stress, sleep, etc.) that are now increasingly being understood to influence cognitive functioning^20,68,69^. Collectively, digital data enables both existing constructs to be redefined and the development of new measures to be identified. Patterns of performance, adherence, and incidental data (i.e., misunderstanding instructions, completing on last day of schedule) may all provide insights into cognitive functioning but require large samples and advanced analytics to do so.

### 4.4 ∣ Limitations

This study provides insight towards future opportunities and challenges associated with remote data collection to assess cognitive functioning. many of which were not able to be addressed in the study. This study was limited by the small sample size, which affected the interpretations of any statistical comparisons related to feasibility and precluded any comparisons of cognitive performance based on impairment status or other established clinical markers. Furthermore, the generalizability of the results to more diverse populations is restricted as the sample consisted of participants from a clinical research center who are mostly White and well-educated. However, the relatively homogenous composition of the study sample is likely a reflection of the parent study rather than a function of the study design per se. Conceptually, the remote study design utilized here should enable recruitment of more representative sample, as has been demonstrated in other literature comparing demographic diversity between in-person and remote research studies^70^. Future efforts should target a larger and more diverse sample, assess both mean scores and variability in cognitive performance relative to assessment frequency, and collect data related to potential within-person influences on cognitive function, such as sleep, diet, stress, and physical activity.

## 5 ∣ Contribution to the field

This study provides support for the limited but growing literature that suggests completing high-frequency mobile-based cognitive assessments is feasible for older adults, including those with CI. This study is the first to demonstrate that degree of specificity by which adherence is defined impacts observed completion rates of high-frequency mobile-based cognitive assessments among older adults with and without CI. When adherence was defined narrowly according to specific dates, participants appeared to complete fewer self-administered assessments as assessment frequency lengthened and time participating in the study increased. However, if adherence was considered more broadly to include the total number of prescribed assessments, rather than according to a specific timeframe, completion rates remained high. As self-administered remote assessments become considered a valid approach to measuring cognitive functioning, understanding the patterns of completion becomes critical towards informing future study designs and protocols, as well as participant selection. Furthermore, considering assessment completion patterns may be a useful variable to consider in conjunction with objective cognitive performance to identify early signs of CI.

## Figures and Tables

**Table 1:** Characteristics of sample at study enrollment

Characteristics	Sample (n=33)	
	*Mean*	*SD*
**Age (Years)**	73.5	7.0
**Education (Years)**	16.9	2.1
	*N*	%
**Sex**		
Female	19	57.6
**Race**		
White	27	81.8
Black/African American	6	18.2
**Personal Mobile Device**		
iPhone	19	57.6
Android	11	33.3
iPad	3	9.1
**Cognitive Status**		
Cognitively Unimpaired	24	72.7
Cognitively Impaired	9	27.3

*Note:* SD = standard deviation.

**Table 2:** Adherence rates across segments and adherence types

	Subsegment Adherence^a^	Segment Adherence^b^	Cumulative Adherence
Segment 1	31/33 (93.9%)	31/33 (93.9%)	-
Segment 2	30/33 (90.9%)	32/33 (97.0%)	-
Segment 3	27/33 (81.8%)	32/33 (97.0%)	-
Segment 4	24/33 (72.7%)^c^	29/33 (87.9%)	-
**Overall**	20/33 (60.6%)	26/33 (78.8%)	30/33 (90.9%)

a Cochran’s Q Test, p = 0.04

b Cochran’s Q Test, p = 0.35

c Pairwise McNemar’s Test for Segment 1 and Segment 4 comparison adjusted p = 0.05

## Abbreviations

(AD): Alzheimer’s disease
(NP): neuropsychological
(CI): cognitive impairment
(MCI): mild cognitive impairment
(IIV-D): intraindividual variability-dispersion
(IIV-I): intraindividual variability-inconsistency
(ADRC): Boston University Alzheimer’s Disease Research Center
(NACC): National Alzheimer’s Coordinating Center
(UDS): Uniform Data Set
(SRT): Simple Reaction Time
(PRT): Procedural Reaction Time
(GNG): Go/NoGo
(CE): Cognitive Efficiency
(BPA): Boston Process Approach
